# Family Caregivers’ Decision-Support Needs Beyond the Decision Aid

**DOI:** 10.1093/geroni/igaa057.219

**Published:** 2020-12-16

**Authors:** Katherine Britt, Karen Schlag

**Affiliations:** 1 The University of Texas at Austin, Austin, Texas, United States; 2 University of Texas at Austin, Austin, Texas, United States

## Abstract

Of the estimated 16 million U.S. family members currently providing essential yet unpaid caregiving for persons with dementia (PWD), many will also make end-of-life (EOL) care decisions as surrogates, a process that can be fraught with uncertainty. Even with dementia death rates rising, many families delay advanced care planning (ACP) discussions, and surrogate decision makers often lack crucial information and support, implicating the need to further study this topic in aging. While decision aids (DA) serve as a support tool for caregivers, they can be less effective when failing to address unresolved decisional needs. Utilizing the Ottawa Decision Support Framework (ODSF), which asserts caregiver decision needs affect decision quality, this study sought to identify surrogate decision-support needs extending beyond general ACP. This mixed study used cognitive interviews and focus groups with family caregivers (N=13) and healthcare professionals (n=14) to assess their knowledge and understanding of hospice and artificial hydration and nutrition. Data were audio-recorded, transcribed verbatim, and analyzed with thematic content analysis. Three main themes were identified: DAs alone aren’t enough to foster quality decision making for surrogates; individualized communication is necessary to clarify PWD and caregiver value priorities and disease trajectories; and clarification of the impact of care choices within situational contexts is quintessential. Further development is needed to create a practice protocol from these themes to inform professionals assisting surrogates in ACP at EOL. Practical implications from this study include highlighting the importance of individualized communication between PWD, providers, and caregivers in addressing EOL care decisional needs.

